# Visibility graphs for fMRI data: Multiplex temporal graphs and their modulations across resting-state networks

**DOI:** 10.1162/NETN_a_00012

**Published:** 2017-10-01

**Authors:** Speranza Sannino, Sebastiano Stramaglia, Lucas Lacasa, Daniele Marinazzo

**Affiliations:** Department of Data Analysis, Faculty of Psychology and Educational Sciences, University of Ghent, Belgium; Department of Physics, University of Bari and INFN Section of Bari, Italy; School of Mathematical Sciences, Queen Mary University of London, United Kingdom; Department of Electric and Electronic Engineering, University of Cagliari, Italy

**Keywords:** Multivariate visibility graphs, Multiplex networks, Resting state fMRI

## Abstract

Visibility algorithms are a family of methods that map time series into graphs, such that the tools of graph theory and network science can be used for the characterization of time series. This approach has proved a convenient tool, and visibility graphs have found applications across several disciplines. Recently, an approach has been proposed to extend this framework to multivariate time series, allowing a novel way to describe collective dynamics. Here we test their application to fMRI time series, following two main motivations, namely that (a) this approach allows vs to simultaneously capture and process relevant aspects of both local and global dynamics in an easy and intuitive way, and (b) this provides a suggestive bridge between time series and network theory that nicely fits the consolidating field of network neuroscience. Our application to a large open dataset reveals differences in the similarities of temporal networks (and thus in correlated dynamics) across resting-state networks, and gives indications that some differences in brain activity connected to psychiatric disorders could be picked up by this approach.

## Author Summary

Here we present the first application of multivariate visibility graphs to fMRI data. Visibility graphs are a way to represent a time series as a temporal network, evidencing specific aspects of its dynamics, such as extreme events. Multivariate time series, as those encountered in neuroscience, and in fMRI in particular, can be seen as a multiplex network, in which each layer represents a time series (a region of interest in the brain in our case). Here we report the method, we describe some relevant aspects of its application to BOLD time series, and we discuss the analogies and differences with existing methods. Finally, we present an application to a high-quality, publicly available dataset, containing healthy subjects and psychotic patients, and we discuss our findings. All the code to reproduce the analyses and the figures is publicly available.

Visibility graphs were recently introduced as a method to map time series into networks (Lacasa, Luque, Ballesteros, Luque, & Nuño, 2008; Luque, Lacasa, Ballesteros, & Luque, 2009), with the aims of using the tools of network science (Boccaletti et al., 2014; Newman, 2010) to describe the structure of time series and their underlying dynamics. This strategy of transforming time series into graphs has been exploited in recent years by some authors and several alternative methods have been put forward, contributing to the nascentfield of performing graph theoretical time series analysis (see Donner, Zou, Donges, Marwan, & Kurths, 2010; Xu, Zhang, & Small, 2008; Zhang & Small, 2006, for a few seminal examples and Gao, Small, & Kurths, 2016 and references therein for a recent overview). Research on visibility graphs has since then focused essentially on two separated avenues. First, analytic studies have primarily explored the foundations of this mapping (Gutin, Mansour, & Severini, 2011; Iacovacci & Lacasa, 2016; Lacasa, 2016; Luque & Lacasa, n.d.) and elaborated on mathematical methods (Lacasa, 2014) to extract rigorous results on the topology of visibility graphs associated to canonical dynamics such as stochastic or chaotic processes (Brú, Gómez-Castro, & Nuño, 2017; Gonçalves, Carpi, Rosso, & Ravetti, 2016; Lacasa, Luque, Luque, & Nuño, 2009; Luque, Ballesteros, Núñez, & Robledo, 2013; Luque, Lacasa, Ballesteros & Robledo, 2011) and to obtain combinatoric analogues of different dynamical quantities (Lacasa, Nuñez, Roldán, Parrondo, & Luque, 2012). The second avenue deals with applications of this machinery, primarily by using this method as a feature extraction procedure with which to build feature vectors that can properly characterize time series with the purpose of making statistical learning (see Bhaduri & Ghosh, 2016; Hou, Li, Wang, & Yan, 2016; Long, Fonseca, Aarts, Haakma, & Foussier, 2014; Shao, 2010, for a few examples in the life sciences). A visibility graph is a network in which the nodes are time points, and the links are defined according to the visibility criteria described in the text. In this latter context, the application to neuroscience is in its infancy and has been essentially limited so far to the analysis of electroencephalogram (EEG) data (see Ahmadlou, Adeli, & Adeli, 2010, 2012; Ahmadlou, Ahmadi, Rezazade, & Azad-Marzabadi, 2013; Bhaduri & Ghosh, 2016; Mira-Iglesias, Conejero, & Navarro-Pardo, 2016 for a few examples). The study of fMRI recordings under this lens has been scarce, and in this work we would like to motivate and justify why we think this is a promising enterprise, both from a univariate and—perhaps more interestingly— from a multivariate time series perspective (Lacasa, Nicosia, & Latora, 2015). Multilayer networks incorporate multiple types of interactions between the same nodes. This means that multivariate time series can be represented in a multilayer visibility graph. Among other strategies to map time series intro graphs, using the repertoire of visibility graphs is particularly interesting, not just because its current application is scarce, but also because these methods are well suited to handle the specificities of fMRI data. More concretely, these methods have been shown to be efficient in extracting information and dealing with (a) data polluted with noise (Lacasa et al., 2009), (b) multivariate (Nicosia & Latora, 2015), and (c) non stationary time series (Luque et al., 2009). In order to showcase the usefulness of visibility graphs in neuroscience we will choose a biggish, high-quality public dataset of resting-state fMRI data (Poldrack et al., 2016), and will make use of the family of visibility algorithms to build a multilevel graph of temporal networks, where each node represents a time point, and two nodes are connected if they are *visible* to each other, according to the algorithm explained below. In the case of multivariate time series—as the ones acquired in neuroimaging—each of these networks is actually the layer of a *multiplex* network (usually associated with a recording in a different region of interest (ROI). Being able to integrate all the data in a single structure enables both the intralayer (univariate) and the interlayer (multivariate) analysis simultaneously. We will show that a direct analysis of this network provides genuine and nontrivial information on fMRI data, potentially including the description and possible noninvasive classification of some brain diseases.

## MATERIALS AND METHODS

### fMRI data

We used the public dataset described in Poldrack et al. (2016). These data were obtained from the OpenfMRI database, with accession number ds000030. We use resting-state fMRI data from 121 healthy controls, 50 individuals diagnosed with schizophrenia, 49 individuals diagnosed with bipolar disorder, and 40 individuals diagnosed with ADHD (attention-deficit/ hyperactivity disorder). The demographics are reported in the original paper, and they can additionally be found in the GitHub page containing the results of this study (Marinazzo, 2017a).

The fMRI data were preprocessed with FSL (FMRIB Software Library v5.0). The volumes were corrected for motion, after which slice timing correction was applied to correct for temporal alignment. All voxels were spatially smoothed with a 6 mm FWHM (full width at half maximum) isotropic Gaussian kernel and after intensity normalization, a band pass filter was applied between 0.01 and 0.08 Hz. In addition, linear and quadratic trends were removed. We next regressed out the motion time courses, the average cerebrospinal fluid signal, and the average white matter signal. Global signal regression was not performed. Data were transformed to the MNI152 template, such that a given voxel had a volume of 3 mm × 3 mm × 3mm. Finally, we averaged the signal in 278 ROIs using the template described in Shen, Tokoglu, Papademetris, & Constable (2013). In order to localize the results within the intrinsic connectivity network of the resting brain, we assigned each of these ROIs to one of the nine resting-state networks (seven cortical networks, plus subcortical regions and cerebellum) as described in Yeo et al. (2011).

### Construction of the visibility graphs

The procedure to build up a visibility graph is extensively and clearly described in Lacasa et al. (2008, 2009, 2012) for univariate and Lacasa et al. (2015) for multivariate time series. Here we will recall the basic steps and provide a visualization of the application of the methodology to BOLD data.

Given a time series of *N* data, any two time points *i* and *j* in which the measured quantity takes the values *y*_*i*_ and *y*_*j*_, respectively, will have visibility and consequently will become two connected nodes in the associated *natural visibility* graph if any other data point *y*_*k*_ placed between them fulfills the following condition:

$$y_{k}<y_{i}+(y_{j}-y_{i})\frac{k-i}{j-i}.$$

Together with this convexity criterion, named *natural visibility*, an ordering criterion, named *horizontal visibility*, has also been defined (Lacasa et al., 2009). According to the latter, two time points *i* and *j*, in which the measured quantity takes the values *y*_*i*_ and *y*_*j*_, respectively, will now have horizontal visibility if any other data point *y*_*k*_ placed between them is smaller; that is,

$$y_{k}<\inf\{y_{i},y_{j}\},\;\forall k:i<k<j.$$

**Figure 1.**
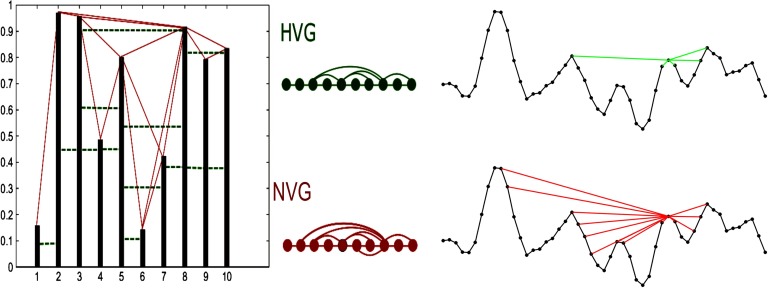
Examples of natural visibility graph (NVG, bottom) and horizontal visibility graph (HVG, top) algorithms applied to the same sample time series. In each case, a time series of *N* data map into a graph of *N* nodes, where two nodes are linked according to either *natural* or *horizontal* visibility criteria (i.e., convexity and ordering criteria, respectively; see the text). On the right side, an illustration of the points connected according to either criterion to a given time point from a typical fMRI region of interest time series.

In either case, the resulting graphs have *N* nodes, are connected by a trivial Hamiltonian path that induces a natural ordering in the degree sequence, and are undirected (see Figure 1 for an illustration). In the event that the time arrow turns out to be a relevant aspect, directed graphs can be easily constructed, as detailed in Lacasa et al. (2012). Note that the resulting horizontal visibility graph (HVG) is simply a core subgraph of the natural visibility graphs (NVG), the former being analytically tractable (Lacasa, 2014). As a matter of fact, HVG can be understood as an order statistic (Lacasa & Flanagan, 2015) and therefore filters out any dependency on the series marginal distributions (this is not true for NVG, so in applications where marginal distributions are relevant, one should use NVG rather than HVG).

Both algorithms are fast: naive implementations of NVGs have a runtime complexity O(*N*^2^); however, a divide-and-conquer strategy already reduces it to O(*N* log *N*) (Lan, Mo, Chen,Liu, & Deng, 2015). Naive implementation of HVG is already O(*N* log *N*) in most of the cases of practical interest. Finally, these methods are well suited to handle several degrees of nonstationarity in the associated time series (Lacasa & Flanagan, 2015).

In this work we will be analyzing BOLD data, and for that task we decided to choose NVG over HVG. This is because NVGs are in principle better suited to handle and extract long-range correlations than HVGs, as the former naturally allow for the development of hubs, which will be typically associated with extreme events in the data and can correlate with data at all scales. Correlations in time series are actually inherited in graph space in the degree distribution. It is somewhat easier to find fat-tailed degree distributions in NVGs (which account for hubs with extremely large degrees). On the other hand, HVGs (which have shown to work fine with processes evidencing short-range correlations) typically display exponentially decaying degree distributions; this feature is linked to short-scale visibility, making this method more local.

For illustration, Figure 1 depicts how the links are established in the visibility graph according to both visibility criteria. The code used to compute the visibility graphs is available on GitHub (Marinazzo, 2017b) and it is basically a translation to Matlab of the original visibility scripts in Fortran 90 (see http://www.maths.qmul.ac.uk/lacasa/VG.f90).

**Figure 2.**
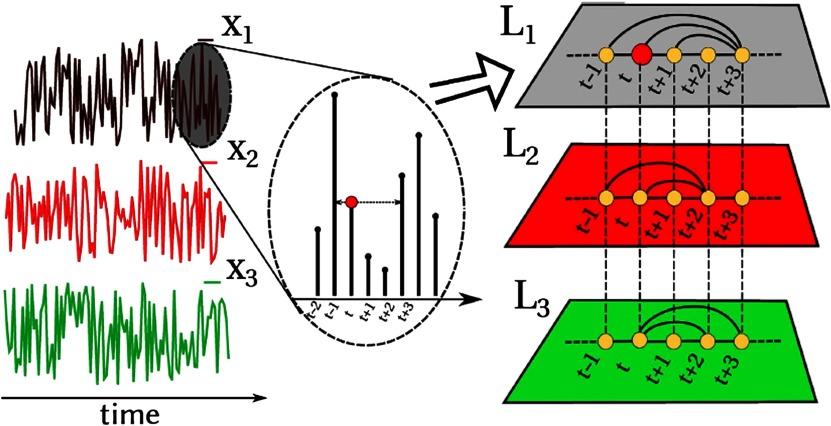
Example of the construction of a multiplex visibility graph from a multivariate time series with *M* = 3 components. In this figure, each layer builds the HVG associated with each variable, therefore, all layers are well aligned according to the time arrow, making interlayer comparison straightforward. Adapted from Lacasa et al. (2015).

When it comes to the application to multivariate time series formed by *M* series, note that each of the *M* time series yields a different visibility graph to begin with, so in principle the multivariate series can always be mapped into a multilayer graph with *M* layers (Lacasa et al., 2015). Moreover, since for every node *i* there is a natural correspondence across layers (node *i* corresponds to time stamp *i*, and this is the same time stamp for all components), there exist a natural alignment between every node of each layer, so the multilayer graph is effectively a so-called multiplex network (Boccaletti et al., 2014; Lacasa et al., 2015, see Figure 2 for an illustration). Of course, other smarter alignments between graphs could be investigated (for instance, one could try to find the alignment that minimizes some sort of Hamming distance between ordered node sets), but in this work we keep it simple and consider the natural alignment induced by the time arrow.

Interestingly, this multiplex visibility graph encodes the complex structure of each time series in the topology of each layer. One can therefore extract in each layer any desired topological feature (say for instance, the entropy over the degree distribution, which would provide a different number for each layer), with which one could build a feature vector that provides a compact representation of the multivariate time series complexity. A similar procedure was followed, for instance, in Ahmadlou et al. (2010) to extract markers of Alzheimer’s disease from a graph theoretical characterization of the Hurst index of EEG data.

Second, the complex interdependencies and correlations that might emerge in a multivariate series across variables could in turn be extracted using similarity measures across layers. There exist a large variety of network measures that one can use for this task (Nicosia & Latora, 2015). A simple example of such a measure is the so-called interlayer mutual information, recently explored in the context of multiplex visibility graphs of coupled chaotic maps Lacasaet al., 2015. This quantity measures the information shared by every two layers based on the similarity of the degree distributions. Given the degree distributions *P*(*k*^*α*^) and *P*(*k*^*β*^) of two arbitrary layers *α* and *β*, it is defined as

$${\text{MI}}_{\alpha ,\beta }=\underset{k^{\left[\alpha \right]}}{\sum }\underset{k^{\left[\beta \right]}}{\sum }P(k^{\left[\alpha \right]},k^{\left[\beta \right]})\log\frac{P(k^{\left[\alpha \right]},k^{\left[\beta \right]})}{P(k^{\left[\alpha \right]})P(k^{[\beta }])}.$$

**Figure 3.**
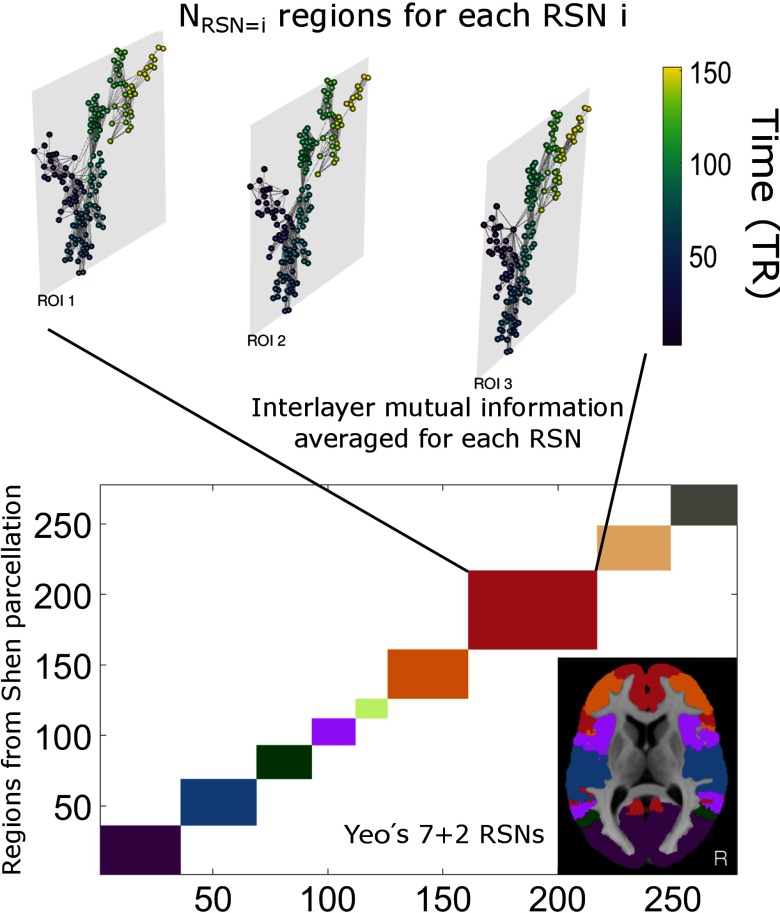
Scheme of the procedure: Within a given region that aggregates a certain number of ROIs, one constructs a visibility graph per ROI and builds accordingly a multiplex visibility graph. We then compute the pairwise mutual information between degree distributions across the multiplex layers (ROIs) and finally average to obtain a value for each RSN (resting-state network). The multilayer network is visualized with MuxViz (De Domenico, Porter, & Arenas, 2015).

As the degree distribution captures the structure of each layer, this measure is in turn capturing the information shared between the two layers, that is, the information shared across each time series component of the multivariate time series. Now, since this is an *M* × *M* matrix whose *ij* entry provides the mutual information between layers (ROIs) *i* and *j*, one can then, for instance, average across pairs (that is, across ROIs) to find a scalar quantity 〈MI〉: the mean value of the mutual information for each intrinsic connectivity network. This methodology is depicted in Figure 3. Note that other informational or similarity measures between layers could be used instead (e.g., edge overlap, conditional or partial mutual information, transfer entropy). Here for the sake of exposition, we consider only mutual information.

**Figure 4.**
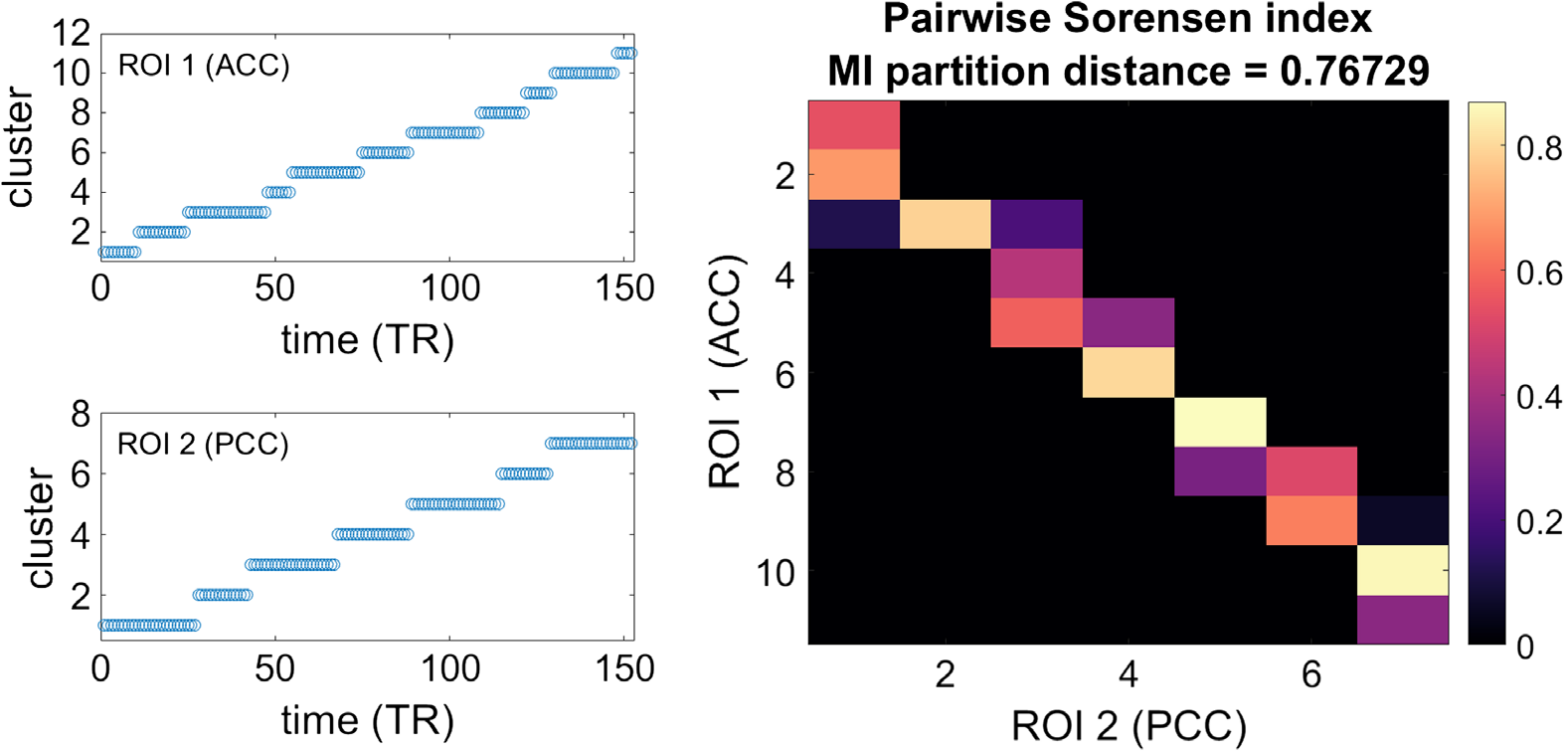
Left: The clusters in which the visibility adjacency matrices from two example ROIs are partitioned according to the Louvain algorithm. Right: Sorensen index quantifying the similarity between pairs of clusters. The value of the distance among the partitioned networks considered as a whole is also reported, in terms of normalized mutual information.

The visibility algorithms produce networks whose nodes are time points. As one can observe in Figure 3, these networks have a modular structure, in which subnetworks are constituted by time points that are mainly adjacent. A network has a modular structure if it can be divided into subnetworks (modules) characterized by a higher probability of connections within each model than across models. A modular structure in a temporal network is thus an indication of different temporal regimes. The existence of these temporal regimes is what motivated the study of dynamical functional connectivity (see, for example, Hansen, Battaglia, Speigler, Deco, &Jirsa, 2013; Hutchison et al., 2013). Dynamic functional connectivity can be seen in the visibility framework as the comparison of the temporal networks, taking their modular structure into account. This comparison can be done in the first place considering the modular network as a whole. In our case we partitioned the visibility graphs for each ROI using 100 runs of the Louvain algorithm. We then quantified the distance between the two partitions by means of the mutual information, using the function in the Brain Connectivity Toolbox (Rubinov & Sporns, 2010). The results of the partition of two ROIs, one in the anterior cingulate cortex (ACC) and one in the precuneus (PCC), are shown in Figure 4. The modules of the graphs correspond to consecutive time points (left panels); that is partitioning the visibility graph provides a natural decomposition of the time series in time intervals. Turning to the interdependency between the two time series, the right panel of Figure 4 represents the Sorensen similarity between each pair of modules in the two time series. It shows that there are segments with high Sorensen indexes, and it is likely that during these segments the two ROIs reflect similar neural events.

## RESULTS

**Figure 5.**
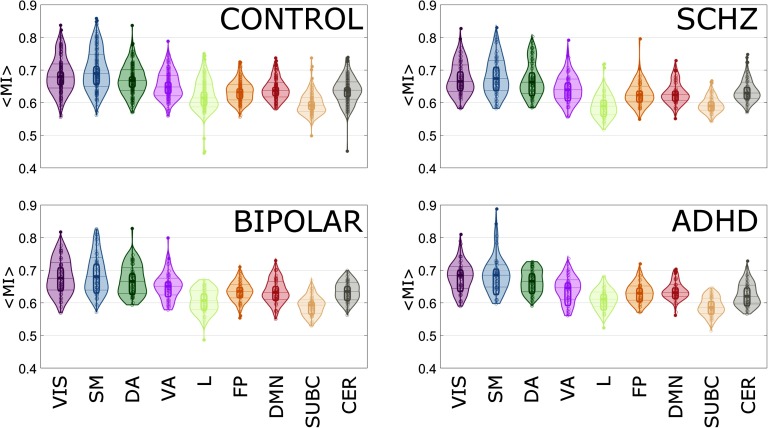
For each group and each intrinsic connectivity network, we plot the distribution across subjects of the averaged interlayer mutual information.

We start by reporting in Figure 5 the results of 〈MI〉 within each of the intrinsic connectivity networks, for the four groups of subjects considered. For each group of subjects, each circle corresponds to 〈MI〉 of a given subject, and random average shifted histograms are also provided. This representation is not parametric, and it is bounded. The plots report the median of the Harrell-Davis estimator, and the 95% high density intervals using a Bayesian bootstrap. The Harrell-Davis estimator doe is independent of the distribution (nonparametric) and is a weighted linear combination of order statistics.

The outliers are detected based on the distance between each pair of data points without assuming symmetry of distributions.

In order to account for departure from normality of these distributions, we used a graphical approach and computed the Kolmogorov-Smirnov distance (Rousselet, Pernet, & Wilcox, 2017), obtaining values up to 0.7 (a value of 0.39 would correspond to rejecting the null hypothesis at a level *α* < 0.001 for the smallest population). The Kolmogorov-Smirnov statistic is a nonparametric test of the equality of continuous, one-dimensional probability distributions.

The number of ROIs constituting each intrinsic state network (thus a proxy for the network size, given that Shen’s parcellation has ROIs of similar size) is not correlated with the average value of the mutual information. In particular, it is interesting to observe that the intrinsic connectivity network called limbic in Yeo’s parcellation is the smallest one, but nonetheless it has a low interlayer mutual information compared with the other networks for all the clinical groups.

**Figure 6.**
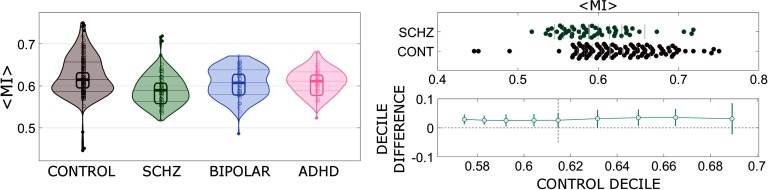
Left: The average interlayer mutual information for the intrinsic connectivity network denoted as limbic, for the four groups of subjects. Right: Shift function to visually and statistically compare the distributions for controls and schizoprenics, at different quantiles.

The network that showed the clearest differentiation in terms of the average interlayer mutual information among the four clinical groups is indeed the Limbic one (Figure 6). This evidence was assessed by means of a multivariate response test with age of the subjects and framewise displacement as covariates. The *p* value of 0.005 was corrected for multiple comparisons using the Bonferroni-Holm criterion with *α* = 0.05. The Kolmogorov-Smirnov statistics of the pairwise comparison between the distributions of average interlayer mutual information values for these particular networks ranged from 0.15 to 0.3. The null hypothesis of values for controls and schizophrenics drawn from the same distribution would be rejected with an *α* < 0.005. Figure 6 also reports the shift functions to visualize the difference between two distributions, in this case controls and schizophrenics. The shift function can help us understand and quantify how the two distributions differ. The shift function describes how one distribution should be rearranged to match the other one: it estimates how and by how much one distribution must be shifted.

This function (Wilcox, 1995) does not assume (as *t* tests do) that two distributions differ only in the location of the bulk of the observations, and it enables determination of how, and by how much, two distributions differ. Here the Harrell-Davis quantile estimator is used. Confidence intervals of the decile differences with a bootstrap estimation of the standard error of the deciles are computed, and one controls for multiple comparisons so that the type I error rate remains around 0.05 across the nine confidence intervals (Rousselet et al., 2017). In this specific case we can observe a clear separation for all the quantiles but the ninth one.

To complement this analysis, in Figure 1 we further report two additional ways in which results of this kind are often represented (mean and standard errors). According to this plot, it is already evident to the naked eye that the method easily distinguishes controls from patients with any mental disorder, suggesting that visibility graphs do indeed extract informative features that can be used for noninvasive diagnosis. Visualizing results in such a way is indeed suboptimal and sometimes problematic (nicely explained in Rousselet, Foxe, & Bolam, 2016); for this reason we initially chose the visualizations provided in Figures 5 and 6 (Rousselet et al., 2017).

## DISCUSSION

### Why the (Multivariate) Visibility Graph?

All in all, there are several reasons why we think that visibility graphs are a convenient tool. We discuss some of these reasons below.

#### Usefulness

Visibility graphs have been shown to inherit in their topology the essence of the associated dynamics, including nontrivial fingerprints that are both descriptive and informative for statistical learning purposes.

#### Fit for purpose

These methods can be used directly in both stationary and nonstationary signals (i.e., nonstationarity is not required to be removed). Also, series do not require ad hoc phase partitioning or symbolization. Also, visibility graphs naturally filter out linear trends, so they do not require such detrending (Lacasa et al., 2008). Furthermore, since HVG is an order statistic, it is also invariant under monotonic (order-preserving) rescaling on the data (Lacasa & Flanagan, 2015). The NVG is not invariant under this latter transformation however, so nonlinear rescaling to make data more “peaky” will necessarily modify the associated NVG in a nontrivial way.

#### Computationally easy and efficient

The method is numerically straightforward to implement and the runtime algorithms are quite decent, varying from *O*(*N*) for so-called visibility sequential motifs (Iacovacci & Lacasa, 2016) to O(*N* log *N*) for the full adjacency matrices using a divide-and-conquer strategy.

#### Amenable to analytical insight

Unlike other strategies for graph theoretical time series analysis, visibility graphs are not computational black boxes. More particularly for HVG (but not only Iacovacci & Lacasa, 2016; Luque et al., 2009), there exist several theorems available and methods to build rigorous results of HVG properties (Lacasa, 2014, 2016; Lacasa et al., 2009, 2012; Luque et al., 2013). The latter is an area of intense research activity at the interface between combinatorics and dynamical systems.

#### Versatile

The methods are not context dependent but are generally applicable to both univariate and multivariate time series across the disciplines. A drawback of this property is that the topological features one can extract from these graphs are themselves not context dependent.

#### Novel

It builds a bridge between time series and networks and thus opens the exciting possibility of exploring the usefulness of a large set of new tools in the endeavor to describe and classify complex signals.

Coming back to the specific reason why we think that natural visibility graphs are particularly suited for BOLD data, it has been shown that relevant information on the time course of the BOLD signal and on correlated activity can be extracted by looking at single frames, corresponding to peaks in the signal (Liu & Duyn, 2013; Tagliazucchi, Balenzuela, Fraiman, & Chialvo, 2012), and that these events could be the proxy for an innovation signal at the neural level (Karahanoglu & Van De Ville, 2015; Wu et al., 2013). In this framework, the degree of the nodes corresponding to the BOLD peaks in the adjacency matrix constructed according to natural visibility emphasizes the functional relevance of the neural events and of the cor responding patterns of coactivation across the brain. However, both NVG and HVG have been shown to be useful in different contexts, so there is no general rule of thumb on what method should we use: this choice shall be addressed on a case-by-case basis.

Finally, what is important and informative when describing the properties of a certain cognitive state? Is it the complex pattern underlying the structure of individual time series (that is, local activity of ROIs) of different regions? Or are the correlations and interdependencies (understood in a broad sense) between these regions the key aspect to look at? When the latter is the case, a functional network analysis approach (Bullmore & Sporns, 2009) seems appropriate. In the former case where the nature of local activity across regions already captures information (He, 2011; Zang, Jiang, Lu, He, & Tian, 2004), one does not need to resort to functional dependencies and local analysis is the correct thing to do. This is obviously an open question that should be addressed, from a biological point of view, on a case-by-case basis. A recent study suggests that both conceptual frameworks can indeed be connected (Sethi, Zerbi, Wenderoth, Fornito, & Fulcher, 2017). In general, both aspects likely play a relevant role, and some studies have already successfully merged the two (Ciuciu, Abry, & He, 2014; Tagliazucchi et al., 2016). Nevertheless, the multiplex visibility framework offers a compact way of extracting at once both the local temporal structure (via the network intra layer properties) and the global interconnection pattern (via multiplex interlayer similarities).

### Similarity with other measures

We discussed at the end of the Methods section that the modular temporal graphs resulting from the visibility algorithm are a natural way to describe different dynamical regimes of individual time series, and their interdependence, without arbitrary and possibly problematic choices such as a sliding window and its length (Hindriks et al., 2016; Kudela, Harezlak, & Lindquist, 2017).

Features of the visibility graph, such as the modularity, the clustering coefficient, or the node degree, could be used as features in classification algorithms aimed to detect modulations of the local and correlated dynamical regime of BOLD signals.

Furthermore, using the excellent resource NeuroVault (http://neurovault.org/), we also looked at the maps depicting the results of other measures and noticed that the areas belonging to the limbic Yeo network are associated with lower levels of regional homogeneity (Zang et al., 2004), higher coefficient of variation of the BOLD signal (Wu & Marinazzo, 2016), and lower value of the fractional amplitude of low-frequency fluctuations (fALFF) (Zou et al., 2008). This evidence speaks to the fact that interlayer mutual information in multiplex visibility networks is associated with decreased predictability and increased independence between the degrees of freedom of the measured time series.

### Classification of neural disorders

The main focus of this paper is methodological, and a thorough discussion of the implications of our results on neuroimaging studies of psychiatric disorders is beyond its scope; moreover, we would not want to hypothesize after the results are known (HARKing) (Poldrack et al., 2017). However, it is interesting to highlight that the limbic network has been previously associated with mental disorders (Kiehl, 2006; Liston, Cohen, Teslovich, Levenson, & Casey, 2011; Potvin, Lungu, Tikàsz, & Mendrek, 2017; Rdulescu & Mujica-Parodi, 2009; Roberts et al., 2016; Whalley et al., 2012). In the same way, we refer the reader to recent studies specifically aimed at using advanced neuroimaging data analysis tools to map and classify neural disorders (Cetin et al., 2016; Demirci et al., 2008; Miller, Vergara, Keator, & Calhoun, 2016), and Fornito, Zalesky, & Breakspear (2015) for a review. Our results shown here using visibility graphs confirm some of this previous work and further showcase that visibility graphs extract informative features with which we can find statistically significant signatures of different neural disorders.

To conclude, given the exposition and results reported in this study, we hope to have motivated our colleagues to consider visibility graphs as a valuable tool for both exploratory and focused studies.

## ACKNOWLEDGMENTS

We thank Matteo Fraschini (University of Cagliari) for setting up the Erasmus mobility for Speranza. We thank Caroline Garcia Forlim for consulting on the mutual information code. We thank Enzo Nicosia (Queen Mary University of London) for stimulating discussions on visibility graphs.

## AUTHOR CONTRIBUTIONS

Speranza Sannino: Formal analysis; Software; Writing original draft. Sebastiano Stramaglia: Conceptualization; Methodology; Software; Writing review & editing. Lucas Lacasa: Conceptualization; Software; Writing original draft; Writing review & editing. Daniele Marinazzo: Conceptualization; Investigation; Methodology; Project administration; Software; Supervision; Validation; Visualization; Writing original draft; Writing review & editing.

## TECHNICAL TERMS

Visibility graph: A visibility graph is a network in which the nodes are time points, and the links are defined according to the visibility criteria described in the text.
Multilayer networks: Multilayer networks incorporate multiple types of interactions between the same nodes. This means that multivariate time series can be represented in a multilayer visibility graph.
Harrell-Davis estimator: The Harrell-Davis estimator does is independent of the distribution (nonparametric) and is a weighted linear combination of order statistics.
Kolmogorov-Smirnov statistic: The Kolmogorov-Smirnov statistic is a nonparametric test of the equality of continuous, one-dimensional probability distributions.
Shift function: The shift function can help us understand and quantify how the two distributions differ. The shift function describes how one distribution should be rearranged to match the other one: it estimates how and by how much one distribution must be shifted.
Modularity in networks: A network has a modular structure if it can be divided into subnetworks (modules) characterized by a higher density of connections within each module than across modules.

## References

[bib1] Ahmadlou M., Adeli H., & Adeli A. (2010). New diagnostic EEG markers of the Alzheimer’s disease using visibility graph. Journal of Neural Transmission, 117(9), 1099–1109. 10.1007/s00702-010-0450-3 20714909

[bib2] Ahmadlou M., Adeli H., & Adeli A. (2012). Improved visibility graph fractality with application for the diagnosis of autism spectrum disorder. Physica A: Statistical Mechanics and its Applications, 391(20), 4720–4726. 10.1016/j.physa.2012.04.025

[bib3] Ahmadlou M., Ahmadi K., Rezazade M., & Azad-Marzabadi E. (2013). Global organization of functional brain connectivity in methamphetamine abusers. Clinical Neurophysiology, 124(6), 1122–1131. 10.1016/j.clinph.2012.12.003 23332777

[bib4] Bhaduri A., & Ghosh D. (2016). Quantitative assessment of heart rate dynamics during meditation: An ECG based study with multi-fractality and visibility graph. Frontiers in Physiology, 7, 44 10.3389/fphys.2016.00044 26909045 PMC4754439

[bib5] Boccaletti S., Bianconi G., Criado R., del Genio C., Gómez-Gardeñes J., Romance M., … Zanin M. (2014). The structure and dynamics of multilayer networks. Physics Reports, 544(1), 1–122. 10.1016/j.physrep.2014.07.001 PMC7332224 32834429

[bib6] Brú A., Gómez-Castro D., & Nuño J. (2017). Visibility to discern local from nonlocal dynamic processes. Physica A: Statistical Mechanics and its Applications, 471, 718–723. 10.1016/j.physa.2016.12.078

[bib7] Bullmore E., & Sporns O. (2009). Complex brain networks: Graph theoretical analysis of structural and functional systems. Nature Reviews Neuroscience, 10(3), 186–198. 10.1038/nrn2575 19190637

[bib8] Cetin M. S., Houck J. M., Rashid B., Agacoglu O., Stephen J. M., Sui J., … Calhoun V. D. (2016). Multimodal classification of schizophrenia patients with MEG and fMRI data using static and dynamic connectivity measures. Frontiers in Neuroscience, 10, 466 10.3389/fnins.2016.00466 27807403 PMC5070283

[bib9] Ciuciu P., Abry P., & He B. J. (2014). Interplay between functional connectivity and scale-free dynamics in intrinsic fMRI networks. NeuroImage, 95, 248–263. 10.1016/j.neuroimage.2014.03.047 24675649 PMC4043862

[bib58] De Domenico M., Porter M. A., & Arenas A. (2015). MuxViz: A tool for multilayer analysis and visualization of networks. Journal of Complex Networks, 3(2), 159–176. 10.1093/comnet/cnu038

[bib10] Demirci O., Clark V. P., Magnotta V. A., Andreasen N. C., Lauriello J., Kiehl K. A., … Calhoun V. D. (2008). A review of challenges in the use of fMRI for disease classification / characterization and a projection pursuit application from a multi-site fMRI schizophrenia study. Brain Imaging and Behavior, 2(3), 207 226. 10.1007/s11682-008-9028-1 PMC2701746 19562043

[bib59] Donner R. V., Zou Y., Donges J. F., Marwan N., & Kurths J. (2010). Recurrence networks—A novel paradigm for nonlinear time series analysis. New Journal of Physics, 12(3), 033025.

[bib11] Fornito A., Zalesky A., & Breakspear M. (2015). The connectomics of brain disorders. Nature Reviews Neuroscience, 16(3), 159–172. 10.1038/nrn3901 25697159

[bib12] Gao Z.-K., Small M., & Kurths J. (2016). Complex network analysis of time series. EPL (Europhysics Letters), 116(5), 50001 Retrieved from http://stacks.iop.org/0295-5075/116/i=5/a=50001

[bib13] Gutin G., Mansour T., & Severini S. (2011). A characterization of horizontal visibility graphs and combinatorics on words. Physica A, 390(12), 2421–2428.

[bib14] Gonçalves B. A., Carpi L., Rosso O. A., & Ravetti M. G. (2016). Time series characterization via horizontal visibility graph and information theory. Physica A: Statistical Mechanics and its Applications, 464, 93–102. 10.1016/j.physa.2016.07.063

[bib15] Hansen E. C. A., Battaglia D., Spiegler A., Deco G., & Jirsa V. K. (2015). Functional connectivity dynamics: Modeling the switching behavior of the resting state. NeuroImage, 105, 525–535. 10.1016/j.neuroimage.2014.11.001 25462790

[bib16] He B. J. (2011). Scale-free properties of the functional magnetic resonance imaging signal during rest and task.The Journal of Neuroscience, 31(39), 13786–13795. 10.1523/JNEUROSCI.2111-11.2011 21957241 PMC3197021

[bib17] Hindriks R., Adhikari M., Murayama Y., Ganzetti M., Mantini D., Logothetis N., (2016). Can sliding-window correlations reveal dynamic functional connectivity in resting-state fMRI? NeuroImage, 127, 242–256. 10.1016/j.neuroimage.2015.11.055 26631813 PMC4758830

[bib18] Hou F., Li F., Wang J., & Yan F. (2016). Visibility graph analysis of very short-term heart rate variability during sleep. Physica A: Statistical Mechanics and its Applications, 458, 140–145. 10.1016/j.physa.2016.03.086

[bib19] Hutchison R. M., Womelsdorf T., Allen E. A., Bandettini P. A., Calhoun V. D., Corbetta M., … Chang C. (2013). Dynamic functional connectivity: Promise, issues, and interpretations. NeuroImage, 80, 360–378. 10.1016/j.neuroimage.2013.05.079 23707587 PMC3807588

[bib20] Iacovacci J., & Lacasa L. (2016). Sequential motif profile of natural visibility graphs. Physical Review E, 94(5), 052309 10.1103/PhysRevE.94.052309 27967131

[bib21] Karahanoglu F. I., & Van De Ville D. (2015). Transient brain activity disentangles fMRI resting-state dynamics in terms of spatially and temporally overlapping networks. Nature Communications, 6, 7751 10.1038/ncomms8751 PMC4518303 26178017

[bib22] Kiehl K. A. (2006). A cognitive neuroscience perspective on psychopathy: Evidence for paralimbic system dysfunction. Psychiatry Research, 142(2–3), 107–128. 10.1016/j.psychres.2005.09.013 16712954 PMC2765815

[bib23] Kudela M., Harezlak J., & Lindquist M. A. (2017). Assessing uncertainty in dynamic functional connectivity. NeuroImage, 149, 165–177. 10.1016/j.neuroimage.2017.01.056 28132931 PMC5384341

[bib24] Lacasa L. (2014). On the degree distribution of horizontal visibility graphs associated with markov processes and dynamical systems: diagrammatic and variational approaches. Nonlinearity, 27(9), 2063 Retrieved from http://stacks.iop.org/0951-7715/27/i=9/a=2063

[bib25] Lacasa L. (2016). Horizontal visibility graphs from integer sequences. Journal of Physics A: Mathematical and Theoretical, 49(35), 35LT01 Retrieved from http://stacks.iop.org/1751-8121/49/i=35/a=35LT01

[bib60] Lacasa L., & Flanagan R. (2015). Time reversibility from visibility graphs of nonstationary processes. Phys. Rev. E, 92, 022817 10.1103/PhysRevE.92.022817 26382464

[bib26] Lacasa L., Luque B., Ballesteros F., Luque J., & Nuño J. C. (2008). From time series to complex networks: The visibility graph.Proceedings of the National Academy of Sciences of the United States of America, 105(13), 4972–4975. 10.1073/pnas.0709247105 18362361 PMC2278201

[bib61] Lacasa L., Luque B., Luque J., & Nuño J. C. (2009). The visibility graph: A new method for estimating the hurst exponent of fractional Brownian motion. EPL (Europhysics Letters), 86(3), 30001 Retrieved from http://stacks.iop.org/0295-5075/86/i=3/a=30001

[bib27] Lacasa L., Nicosia V., & Latora V. (2015). Network structure of multivariate time series.Scientific Reports, 5, 15508 10.1038/srep15508 26487040 PMC4614448

[bib28] Lacasa L., Nuñez A., Roldán É., Parrondo J. M. R., & Luque B. (2012). Time series irreversibility: A visibility graph approach. European Physical Journal B, 85(6), 217 10.1140/epjb/e2012-20809-8

[bib62] Lan X., Mo H., Chen S., Liu Q., & Deng Y. (2015). Fast trans formation from time series to visibility graphs. Chaos: An Interdisciplinary Journal of Nonlinear Science, 25(8), 083105 10.1063/1.4927835 26328556

[bib29] Liston C., Cohen M. M., Teslovich T., Levenson D., & Casey B. (2011). Atypical prefrontal connectivity in attention-deficit/ hyperactivity disorder: Pathway to disease or pathological end point? Biological Psychiatry, 69(12), 1168–1177. 10.1016/j.biopsych.2011.03.022 21546000

[bib63] Liu X., & Duyn J. H. (2013). Time-varying functional network information extracted from brief instances of spontaneous brain activity.Proceedings of the National Academy of Sciences of the United States of America, 110(11), 4392–4397. 10.1073/pnas.1216856110 23440216 PMC3600481

[bib64] Long X., Fonseca P., Aarts R. M., Haakma R., & Foussier J. (2014). Modeling cardiorespiratory interaction during human sleep with complex networks. Applied Physics Letters, 105(20), 203701 10.1063/1.4902026

[bib30] Luque B., Ballesteros F. J., Núñez A. M., & Robledo A. (2013). Quasiperiodic graphs: Structural design, scaling and entropic properties. Journal of Nonlinear Science, 23(2), 335–342. 10.1007/s00332-012-9153-2

[bib31] Luque B., & Lacasa L. (n.d.). Canonical horizontal visibility graphs are uniquely determined by their degree sequence. arXiv: 1605.05222.

[bib65] Luque B., Lacasa L., Ballesteros F., & Luque J. (2009). Horizontal visibility graphs: Exact results for random time series. Phys. Rev. E, 80, 046103 10.1103/PhysRevE.80.046103 19905386

[bib66] Luque B., Lacasa L., Ballesteros F. J., & Robledo A. (2011). Feigenbaum graphs: A complex network perspective of chaos. PLoS ONE, 6(9), 1–8. 10.1371/journal.pone.0022411 PMC3168432 21915254

[bib32] Marinazzo D. (2017a). Visibility_LA5C_data. GitHub reposi tory. Retrieved from https://github.com/danielemarinazzo/Visibility_LA5C_data

[bib33] Marinazzo D. (2017b). Visibility. GitHub repository. Retrieved from https://github.com/danielemarinazzo/Visibility

[bib34] Miller R. L., Vergara V. M., Keator D. B., & Calhoun V. D. (2016). A method for intertemporal functional-domain connectivity analysis: Application to schizophrenia reveals distorted directional information flow. IEEE Transactions on Biomedical Engineering, 63(12), 2525–2539. 10.1109/TBME.2016.2600637 27541329 PMC5737021

[bib67] Mira-Iglesias A., Conejero J. A., & Navarro-Pardo E. (2016). Natural visibility graphs for diagnosing attention deficit hyperactivity disorder (ADHD). Electronic Notes in Discrete Mathematics, 54, 337–342. 10.1016/j.endm.2016.09.058

[bib35] Newman M. (2010). Networks: An introduction. Oxford: Oxford University Press.

[bib36] Nicosia V., & Latora V. (2015). Measuring and modeling cor relations in multiplex networks. Phys. Rev. E, 92, 032805 10.1103/PhysRevE.92.032805 26465526

[bib37] Poldrack R., Congdon E., Triplett W., Gorgolewski K., Karlsgodt K., Mumford J., … Bilder R. (2016). A phenome-wide examination of neural and cognitive function. Scientific Data, 3, 160110 10.1038/sdata.2016.110 27922632 PMC5139672

[bib38] Poldrack R. A., Baker C. I., Durnez J., Gorgolewski K. J., Matthews P. M., Munafò M. R., … Yarkoni T. (2017). Scanning the horizon: Towards transparent and reproducible neuroimag ing research. Nature Reviews Neuroscience, 18(2), 115–126. 10.1038/nrn.2016.167 28053326 PMC6910649

[bib39] Potvin S., Lungu O., Tikàsz A., & Mendrek A. (2017). Abnormal effective fronto-limbic connectivity during emotion processing in schizophrenia. Progress in Neuro-Psychopharmacology and Biological Psychiatry, 72, 1–8. 10.1016/j.pnpbp.2016.08.004 27528110

[bib40] Rdulescu A. R., & Mujica-Parodi L. R. (2009). A principal component network analysis of prefrontal-limbic functional magnetic resonance imaging time series in schizophrenia patients and healthy controls. Psychiatry Research: Neuro imaging, 174(3), 184–194. 10.1016/j.pscychresns.2009.04.017 PMC2788080 19880294

[bib41] Roberts G., Perry A., Lord A., Frankland A., Leung V., Holmes-Preston E., … Breakspear M. (2016). Structural dysconnectivity of key cognitive and emotional hubs in young people at high genetic risk for bipolar disorder. Molecular Psychiatry. Advance online publication. 10.1038/mp.2016.216 PMC5794888 27994220

[bib42] Rousselet G. A., Foxe J. J., & Bolam J. P. (2016). A few simple steps to improve the description of group results in neuroscience. European Journal of Neuroscience, 44(9), 2647–2651. 10.1111/ejn.13400 27628462

[bib43] Rousselet G. A., Pernet C. R., & Wilcox R. R. (2017). Beyond differences in means: Robust graphical methods to compare two groups in neuroscience. bioRxiv. Retrieved from 10.1111/ejn.13610 28544058

[bib44] Rubinov M., & Sporns O. (2010). Complex network measures of brain connectivity: Uses and interpretations. NeuroImage, 52(3), 1059–1069. 10.1016/j.neuroimage.2009.10.003 19819337

[bib45] Sethi S. S., Zerbi V., Wenderoth N., Fornito A., & Fulcher B. D. (2017). Structural connectome topology relates to regional BOLD signal dynamics in the mouse brain. Chaos, 27, 047405. 28456172 10.1063/1.4979281

[bib46] Shao Z.-G. (2010). Network analysis of human heartbeat dynamics. Applied Physics Letters, 96(7), 073703 10.1063/1.3308505

[bib47] Shen X., Tokoglu F., Papademetris X., & Constable R. T. (2013). Groupwise whole-brain parcellation from resting-state fMRI data for network node identification.NeuroImage, 82, 403–415. 10.1016/j.neuroimage.2013.05.081 23747961 PMC3759540

[bib68] Tagliazucchi E., Balenzuela P., Fraiman D., & Chialvo D. R. (2012). Criticality in large-scale brain fMRI dynamics unveiled by a novel point process analysis. Frontiers in Physiology, 3, 15 10.3389/fphys.2012.00015 22347863 PMC3274757

[bib48] Tagliazucchi E., Chialvo D. R., Siniatchkin M., Amico E., Brichant J.-F., Bonhomme V., … Laureys S. (2016). Large-scale signatures of unconsciousness are consistent with a departure from critical dynamics. Journal of the Royal Society Interface, 13(114). 10.1089/rsif.2015.1027 PMC4759808 26819336

[bib49] Whalley H. C., Papmeyer M., Sprooten E., Lawrie S. M., Sussmann J. E., & McIntosh A. M. (2012). Review of functional magnetic resonance imaging studies comparing bipolar disorder and schizophrenia. Bipolar Disorders, 14(4), 411–431. 10.1111/j.1399-5618.2012.01016.x 22631622

[bib50] Wilcox R. R. (1995). Comparing two independent groups via multiple quantiles. The Statistician, 44(1), 91 10.2307/2348620

[bib51] Wu G.-R., Liao W., Stramaglia S., Ding J.-R., Chen H., & Marinazzo D. (2013). A blind deconvolution approach to recover effective connectivity brain networks from resting state fMRI data. Medical Image Analysis, 17(3), 365–374. 10.1016/j.media.2013.01.003 23422254

[bib52] Wu G.-R., & Marinazzo D. (2016). Sensitivity of the resting-state haemodynamic response function estimation to autonomic nervous system fluctuations. Philosophical Transactions of the Royal Society of London A: Mathematical, Physical and Engineering Sciences, 374(2067). 10.1098/rsta.2015.0190 PMC4822449 27044997

[bib53] Xu X., Zhang J., & Small M. (2008). Superfamily phenomena and motifs of networks induced from time series. Proceedings of the National Academy of Sciences, 105(50), 19601–19605. 10.1073/pnas.0806082105 PMC2604928 19064916

[bib54] Yeo B. T., Krienen F. M., Sepulcre J., Sabuncu M. R., Lashkari D., Hollinshead M., (2011). The organization of the human cerebral cortex estimated by intrinsic functional connectivity. Journal of Neurophysiology, 106(3), 1125–1165. 10.1152/jn.00338.2011 21653723 PMC3174820

[bib55] Zang Y., Jiang T., Lu Y., He Y., & Tian L. (2004). Regional homogeneity approach to fMRI data analysis. NeuroImage, 22(1), 394–400. 10.1016/j.neuroimage.2003.12.030 15110032

[bib56] Zhang J., & Small M. (2006). Complex network from pseudo periodic time series: Topology versus dynamics. Physical Review Letters, 96(23), 238701. 16803415 10.1103/PhysRevLett.96.238701

[bib57] Zou Q.-H., Zhu C.-Z., Yang Y., Zuo X.-N., Long X.-Y., Cao Q.-J., … Zang Y.–F. (2008). An improved approach to detection of amplitude of low-frequency fluctuation (ALFF) for resting-state fMRI: Fractional ALFF. Journal of Neuroscience Methods, 172(1), 137–141. 10.1016/j.jneumeth.2008.04.012 18501969 PMC3902859

